# An Eulogium on Doctors

**Published:** 1879-01

**Authors:** 


### An Eulogium on Doctors.

Union Méd., September 5th: In his report to the Académie Française on the Monthyon Prize, Professor Dumas, the distin-distinguished chemist, thus expresses himself concerning medical practitioners, who daily furnish proof of their humanity, courage, and abnegation: "If virtue consists in absolute devotion to duty, do we not find the most certain signs of this in the repeated traits of courage presented to our admiration by medical practitioners, interpreting the oath of Hippocrates in the most noble sense, exposing their own lives in a struggle without glory, in a combat without illusions, surrounded by patients to approach whom may be mortal? Is the danger uncertain? How numerous, on the contrary, are the examples which attest that in certain affections which are but too common it is imminent. Do you ever find a single practitioner hesitate in the accomplishment of his mission? Never! Whether they are aged and enlightened by the experience of the long past, whether they are starting on their career animated still by all the confidence of youth, whether they are living alone, which might excuse selfishness, or married and fathers of families, which would authorize prudence, they are never found to falter. Long, however, would be the list if it had to furnish the names of all these victims of professional duty, of all these practitioners 'dying in front of the enemy,' as they say at the Ministry of War."—*Louisville Med. News.*

